# Exosome-Transmitted miR-25 Induced by *H. pylori* Promotes Vascular Endothelial Cell Injury by Targeting KLF2

**DOI:** 10.3389/fcimb.2019.00366

**Published:** 2019-10-29

**Authors:** Na Li, Shi-feng Liu, Kai Dong, Gui-chun Zhang, Jing Huang, Zhi-heng Wang, Tong-jian Wang

**Affiliations:** Department of Pediatric Cardiac Surgery, Institute of Cardiovascular Disease, The 960th Hospital of Chinese PLA, Jinan, China

**Keywords:** miR-25, *Helicobacter pylori*, coronary heart disease, NF-κB, KLF2

## Abstract

**Background:** Increasing evidence has shown that *Helicobacter pylori* is associated with coronary heart disease (CHD); however, the underlying mechanism remains unclear.

**Methods:** The expression of miR-25 and mRNAs was measured using qRT-PCR. Protein levels were detected using western blotting and exosomes were assessed with an electron microscope. The target gene of miR-25 was identified using the luciferase report system.

**Results:** *H. pylori* infection increased the expression of miR-25 in gastric epithelial cells and was associated with increased levels of exosome-transmitted miR-25 in human peripheral blood. Mechanistic investigation showed the Kruppel-like factor 2 (KLF2) was a direct target of exosome-transmitted miR-25 in vascular endothelial cells. In addition, the miR-25/KLF2 axis regulated the NF-κB signaling pathway, resulting in increased expression of interleukin 6 (IL6), monocyte chemoattractant protein-1 (MCP-1), vascular cell adhesion molecule-1 (VCAM-1), and intercellular adhesion molecule-1 (ICAM-1).

**Conclusion:** Our findings suggest that the miR-25/KLF2 axis may be a potential therapeutic target for *H. pylori*-associated CHD. Furthermore, high levels of exosome-transmitted miR-25 in peripheral blood may pose a potential risk for CHD.

## Introduction

*Helicobacter pylori* (*H. pylori*) is a Gram-negative bacterium that can colonize the stomach of humans. *H. pylori* infection has been considered one of the major factors in several gastric diseases, such as gastritis, gastric ulcers, and atrophic gastritis with intestinal metaplasia, which is closely related to gastric cancer (Kim and Shin, 2018). However, increasing evidence has revealed the relationship between *H. pylori* infection and other organ-associated diseases, especially atherosclerosis, which is associated with the incidence of coronary heart disease (CHD) (He et al., 2014). Nikolopoulou et al. (2008) reported that patients with CHD have a higher rate of *H. pylori* infection than healthy people, and *H. pylori* infection is associated with a high risk of CHD incidence. *H. pylori* possibly promotes the incidence of atherosclerosis by aggravating metabolic disorders (Xu Z. et al., 2017); however, the underlying mechanism remains to be elucidated.

Exosomes are cystic vesicles with a double-layer membrane and a diameter of 30~100 nm (Tkach and Thery, 2016). Exosomes are released by almost all types of cells and can contain a variety of proteins, lipids, RNAs, and DNAs. They transmit these contents from one cell to another, thereby facilitating crosstalk among cells (Valadi et al., 2007). In the last decade, the important roles of exosome-transmitted miRNAs in the development of many diseases have been confirmed. For example, lymphocyte-derived exosomal miRNAs promote pancreatic β cell death (Guay et al., 2018). Cancer cell-secreted exosomal miR-105 promotes tumor growth via the MYC-dependent metabolic reprogramming of stromal cells (Yan et al., 2018). Much evidence also demonstrates the important roles of miRNAs in regulating atherosclerosis (Schober and Weber, 2016). Exosomal miR-143/145 derived from endothelial cells can control target gene expression in smooth muscle cells, thereby reducing the formation of atherosclerotic lesions (Hergenreider et al., 2012). This suggests that exosomal miRNAs play a role in atherosclerosis.

A large number of studies have revealed the multiple roles of miR-25 in many diseases (Sarkozy et al., 2018), including atherosclerosis (Qi et al., 2015; Maier et al., 2016). Our previous study has shown that a high level of miR-25 is present in the plasma of patients infected with *H. pylori* (Li et al., 2012), suggesting that *H. pylori* may induce an increase in exosomal miR-25 by infecting gastric epithelial cells. Thus, we aimed to determine whether *H. pylori* infection-induced exosomal miR-25 is involved in atherosclerosis.

## Results

### Patients With *H. pylori* Infection Have High Levels of Exosomal miR-25 in Plasma

**Figure 1:**
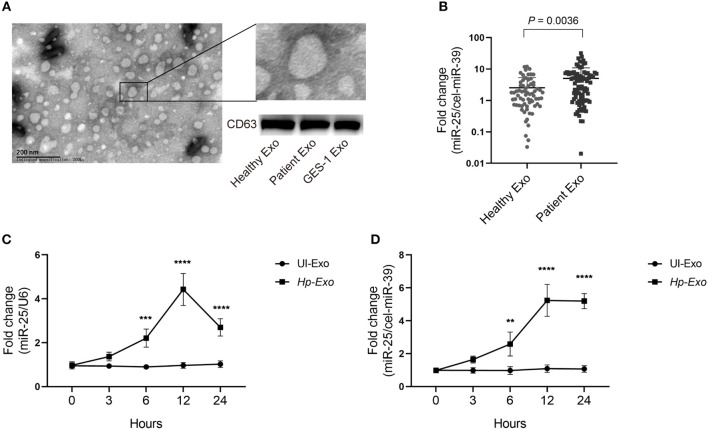
Patients with *H. pylori* infection have high levels of exosomal miR-25 in plasma. **(A)** A representative electron micrograph reveals exosomes isolated from the plasma of patients. **(B)** Expression of miR-25 in exosomes isolated from the plasma of 68 healthy subjects and 86 patients. External *cel-miR-39* was used to normalize miR-25 expression. **(C)** Expression of miR-25 in GES-1 cells at different time points after *H. pylori* infection. **(D)** Expression of miR-25 in exosomes isolated from culture medium of GES-1 cells at different time points after *H. pylori* infection. **P < 0.01; ***P < 0.001; ****P < 0.0001.

To determine whether *H. pylori* infection is associated with exosomal miR-25, we enrolled 86 patients with *H. pylori* infection but without other diseases, and 68 healthy subjects. Exosomes were isolated from plasma samples of both groups. The exosomes were identified using an electron microscope and immunoblotting experiments (Figure 1A). An equal volume of exosomes was used to extract RNAs. We found that levels of exosomal miR-25 were significantly increased in the plasma of patients with *H. pylori* infection, compared with healthy subjects (Figure 1B). As *H. pylori* usually colonizes the gastric mucosa and infects gastric epithelial cells, we used the GES-1 cell line established from the normal gastric epithelium, to analyze whether *H. pylori* regulates the expression of miR-25.

As expected, *H. pylori* infection led to significantly increased levels of miR-25 in GES-1 cells at various times, and miR-25 reached its highest level at 12 h (Figure 1C). Moreover, we isolated exosomes from the cell culture supernatant, and consistent with the results observed in GES-1 cells, the exosomes showed the highest levels of miR-25 at 12 h (Figure 1D). These data suggest that *H. pylori* induces gastric epithelial cell-derived exosomal miR-25.

### Exosome-Transmitted miR-25 Increases Levels of Inflammatory Factors in Endothelial Cells

**Figure 2:**
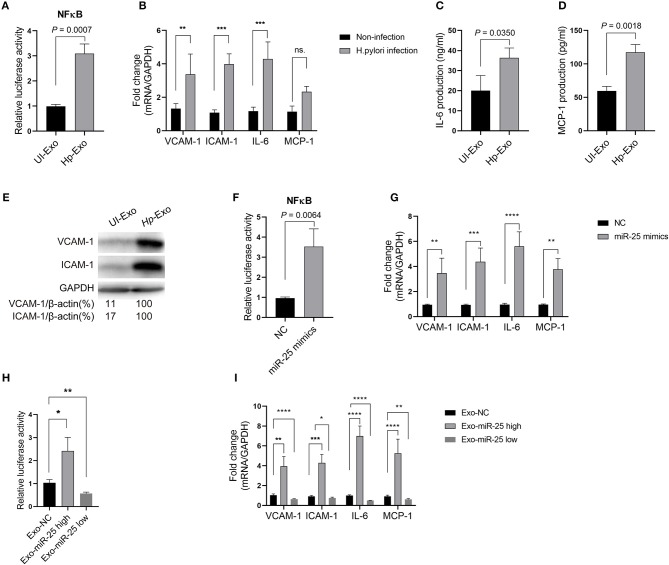
Exosome-transmitted miR-25 increases the levels of inflammatory factors in endothelial cells. **(A)** Bars show relative luciferase activity of NFκB in HEK293 cells treated with exosomes isolated from *H. pylori*-infected or uninfected GES-1 cells. **(B)** Bars show relative mRNA expression of vascular cell adhesion molecule-1 (VCAM-1), intercellular adhesion molecule-1 (ICAM-1), interleukin 6 (IL-6), and monocyte chemoattractant protein-1 (MCP-1) in HUVECs with or without *H. pylori* infection. **(C,D)** Bars show protein levels of IL-6 and MCP-1 released by HUVECs treated with exosomes isolated from *H. pylori*-infected or uninfected GES-1 cells. **(E)** Western blotting shows protein expression of VCAM-1 and ICAM-1 in HUVECs treated with exosomes isolated from *H. pylori*-infected or uninfected GES-1 cells. GAPDH served as the internal reference. **(F)** Bars show relative luciferase activity of NFκB in HEK293 cells transfected with the miR-25 negative control (NC) or miR-25 mimics. **(G)** Bars show mRNA expression of VCAM-1, ICAM-1, IL-6, and MCP-1 in HUVECs transfected with the miR-25 NC or miR-25 mimics. **(H)** Bars show relative luciferase activity of NFκB in HEK293 cells treated with exosomes isolated from GES-1 cells transfected with the miR-25 NC, mimics, and miR-25 inhibitors. **(I)** Bars show mRNA expression of VCAM-1, ICAM-1, IL-6, and MCP-1 in HUVECs treated with exosomes isolated from GES-1 cells transfected with the miR-25 NC, mimics, and miR-25 inhibitors. *P < 0.05; **P < 0.01; ***P < 0.001; ****P < 0.0001.

Atherosclerosis is associated with functional change in the endothelial cells of blood vessels. Thus, we sought to determine whether exosomal miR-25 affects the endothelial cells. Human umbilical vein endothelial cells (HUVECs) were used to perform these experiments. Compared with the exosomes from patients without *H. pylori* infection, we found that exosomes from the plasma of patients with *H. pylori* infection, or from the cell culture supernatant of GES-1 cells with *H. pylori* infection both enhanced the activity of NFκB (Figure 2A) and led to significantly increased levels of vascular cell adhesion molecule-1 (VCAM-1), intercellular adhesion molecule-1 (ICAM-1), interleukin 6 (IL-6), and monocyte chemoattractant protein-1 (MCP-1) in HUVECs (Figures 2B–E).

To determine whether exosome function is dependent on miR-25, its mimics were synthesized and transfected into HUVECs (Figure S1A). We found that miR-25 mimics also significantly enhanced the activity of NF-κB and increased levels of VCAM-1, ICAM-1, IL-6, and MCP-1 in HUVECs (Figures 2F,G). Furthermore, we used miR-25 mimics or inhibitors to increase or repress miR-25 expression in GES-1 cells, respectively, and isolated the exosomes (Figure S1B), which were then used to incubate HUVECs. We found that the exosomes with high or low expression of miR-25 significantly promoted or inhibited the activity of NF-κB and the levels of VCAM-1, ICAM-1, IL-6, and MCP-1 in HUVECs compared with those from normal GES-1 cells (Figures 2H,I). These findings suggest that exosomal miR-25 is a major factor associated with the regulation of inflammatory factor levels in HUVECs.

### KLF2 Is a Direct Target of Exosomal miR-25

**Figure 3:**
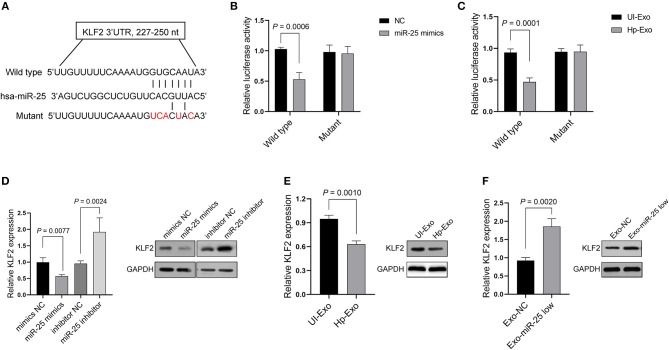
KLF2 is a direct target of exosomal miR-25. **(A)** Binding sequences between miR-25 and the wild-type or mutant 3′UTR of KLF2. **(B,C)** Bars show relative luciferase activity in HEK293 cells transfected with the miR-25 negative control (NC) or miR-25 mimics, or treated with exosomes isolated from GES-1 cells with or without *H. pylori* infection. **(D)** Bars and bands show mRNA and protein expression of KLF2 in HUVECs transfected with a mimic NC, miR-25 mimics, an inhibitor NC, or miR-25 inhibitor. **(E,F)** The mRNA and protein expression of KLF2 in HUVECs treated with exosomes isolated from *H. pylori*-infected or uninfected GES-1 cells, or from GES-1 cells transfected with the miR-25 NC and miR-25 inhibitor.

We sought to elucidate the mechanism by which miR-25 exerts its function in HUVECs. Depending on the base pairing, miRNA can bind to the 3′ untranslated region (UTR) of mRNAs and thereby effect their degradation. Thus, we predicted the candidate target mRNAs that might be the directly regulated by miR-25 using the TarBase v.8 database. The analyses considered Kruppel-like factor 2 (KLF2) as a potential target of miR-25 (Figure 3A), because it has been identified in other cell lines in previous studies and is associated with protection against atherosclerosis. Thus, we initially used the Dual-Luciferase Report Assay System to identify the prediction.

As expected, we found that miR-25 mimics significantly inhibited luciferase activity in HEK293 cells, which were transfected with the vectors containing the wild-type 3′UTR of KLF2 mRNA, but had no influence on the mutant 3′UTR (Figure 3B). Moreover, the exosomes derived from GES-1 cells with *H. pylori* infection played a similar role to that of the miR-25 mimics in HEK293 cells (Figure 3C). Furthermore, miR-25 mimics inhibited the mRNA and protein expression of KLF2, whereas miR-25 inhibitors significantly increased its expression in HUVECs (Figure 3D). Next, we accessed the expression of miR-25 in HUVECs which had incorporated the exosomes derived from GES-1 cells with *H. pylori* infection, and the results showed an significantly increased level of miR-25 in HUVECs (Figure S2). Compared with normal GES-1 cell-derived exosomes, the exosomes derived from GES-1 cells with *H. pylori* infection led to much lower mRNA and protein levels of KLF2 in HUVECs (Figure 3E). Furthermore, the exosomes released by GES-1 cells that were transfected with miR-25 inhibitors significantly increased the mRNA and protein expression of KLF2, compared with those transfected with the miR-25 negative control (NC) (Figure 3F). These data suggest that KLF2 is a direct target of GES-1 cell-derived exosomal miR-25.

### The MiR-25/KLF2 Axis Regulates the NFκB Signaling Pathway

**Figure 4:**
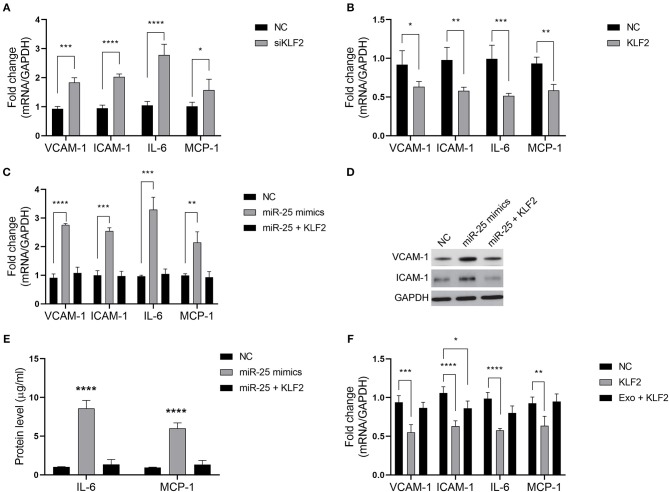
The miR-25/KLF2 axis regulates the NFκB signaling pathway. **(A,B)** Bars show the mRNA expression of vascular cell adhesion molecule-1 (VCAM-1), intercellular adhesion molecule-1 (ICAM-1), interleukin 6 (IL-6), and monocyte chemoattractant protein-1 (MCP-1) in HUVECs transfected with the negative control (NC), KLF2 siRNA (siKLF2), or the KLF2-overexpressing vector (KLF2). **(C)** Bars show the mRNA expression of VCAM-1, ICAM-1, IL-6, and MCP-1 in HUVECs transfected with the NC or miR-25 mimics; or co-transfected with miR-25 (miR-25 mimics) and KLF2; or transfected with the NC or KLF2. **(D,E)** The protein expression of VCAM-1, ICAM-1, IL-6, and MCP-1 in HUVECs transfected with the NC or miR-25 mimics, or co-transfected with miR-25 mimics and KLF2 detected by western blotting and ELISA. **(F)** Bars show the mRNA expression of VCAM-1, ICAM-1, IL-6, and MCP-1 in HUVECs transfected with KLF2 and simultaneously treated with exosomes isolated from GES-1 cells. *P < 0.05; **P < 0.01; ***P < 0.001; ****P < 0.0001.

We sought to determine whether miR-25 regulates the inflammatory factors in HUVECs via KLF2. As previously established, KLF2 affects the activity of NFκB by competitively binding to CBP/p300, resulting in the decreased expression of downstream genes of the NFκB signaling pathway. As expected, KLF2 knockdown significantly increased the levels of VCAM-1, ICAM-1, IL-6, and MCP-1 in HUVECs, whereas ectopic expression of KLF2 significantly inhibited the expression of those molecules (Figures 4A,B).

We found that the expression of VCAM-1, ICAM-1, IL-6, and MCP-1 was rescued when HUVECs were subjected to co-transfection with miR-25 mimics and the KLF2 vector, compared with transfection with miR-25 mimics alone (Figures 4C–E). Moreover, the expression of those molecules that were inhibited by KLF2 was also rescued by GES-1 cell-released exosomes (Figure 4F). These findings suggest that the miR-25/KLF2 axis plays a role in HUVECs by regulating the NFκB signaling pathway.

## Discussion

In the present study, we found that patients with *H. pylori* infection had high levels of exosomal miR-25 in plasma, leading to increased levels of inflammatory factors in endothelial cells through the direct targeting of KLF2, an upstream gene in the NFκB signaling pathway. This mechanism may provide a potentially therapeutic strategy for *H. pylori* infection-associated CHD.

Previous studies have shown that patients with CHD and *H. pylori* infection tend to have worse outcomes (Shan et al., 2018). The underlying reason is considered to be associated with the activation of inflammatory cells within atherosclerotic lesions (Rothwell et al., 2000). *H. pylori* can increase the levels of lipids and fibrinogen during low-grade persistent inflammatory stimulation, as well as anti-heat shock protein 65 (hsp65) antibodies, a hallmark of atherosclerosis (Birnie et al., 1998; Laurila et al., 1999). In the present study, we found that *H. pylori* infection led to increased levels of miR-25 in cell lines of the gastric mucosa, and increased exosomal miR-25 in the plasma of patients. Moreover, we found that exosomal miR-25 significantly increased the expression of inflammatory factors in vascular endothelial cells. Recently, Yao et al. (2018) reported that the expression profile of miR-221, miR-19b-5p, and miR-25-5p in peripheral blood mononuclear cells (PBMCs), and hypertension are the independent predictive factors for heart failure (HF) in CHD patients, and found that the expression of miR-25-5p is significantly increased in PBMCs of CHD patients with HF, suggesting that the increased expression of miR-25-5p is positively correlated with the severity of CHD.

Atherosclerosis is associated with the deposition of lipids in the walls of blood vessels, which causes an inflammatory and proliferative cascade that affects smooth muscle, endothelial, and immune cells (Zhang et al., 2018). Atherosclerosis with concurrent bacterial infection is associated with the aberrant expression of inflammatory factors, which is partially caused by the infection rather than the lipid deposits alone (George et al., 2000; Rothwell et al., 2000). A large number of miRNAs reportedly participate in the regulation of inflammatory factors in endothelial cells (Schober et al., 2015). For example, miR-100 can suppress the expression of several components of mammalian target of rapamycin complex 1-signaling, resulting in leukocyte-endothelial interactions (Pankratz et al., 2018). However, *H. pylori* infection-associated miRNAs involved in atherosclerosis have been rarely investigated.

Our findings revealed that *H. pylori* infection induced an increase in the levels of miR-25 in gastric mucosal cell lines, and exosomal miR-25 in the plasma of patients. This suggests that *H. pylori* infection may influence atherosclerosis via exosomal miR-25. Exosome-transmitted miRNAs play a similar role to that of endogenous miRNAs; however, the main difference is the miRNAs are derived from other cells or organs. Zhou et al. (2014) reported that metastatic breast cancer cell-derived exosomal miR-105 destroys vascular endothelial barriers, thereby promoting metastasis. Similarly, the present results reveal that gastric mucosa-derived exosomal miR-25 gives rise to dysfunctional vascular endothelial cells through an approach of gain- and loss-of-function.

We also found that exosomal miR-25 regulates inflammatory factors in the endothelial cells by targeting KLF2, a repressor of vascular inflammation and atherosclerosis (Xu Y. et al., 2017). As a zinc-finger transcription factor, KLF2 is expressed in the vascular endothelium (Novodvorsky and Chico, 2014), maintains endothelial homeostasis, and controls the expression of multiple genes associated with anti-inflammation in endothelial cells (Lin et al., 2005; Marrone et al., 2013). Furthermore, KLF2 is also involved in the regulation of the NF-κB signaling pathway and the host response to polymicrobial infection (Mahabeleshwar et al., 2011).

Jha and Das (2017) reviewed the crosstalk between KLF2 and NF-κB, which predominantly influences inflammation. SenBanerjee et al. (2004) showed that KLF2 can inhibit the activity of NF-κB by competitively binding to cyclic AMP response element-binding protein (CBP/p300), a cofactor of NF-κB. Consistent with these findings, our results also showed that the miR-25/KLF2 axis regulates inflammatory factors, such as IL6, MCP-1, VACM-1, and ICAM-1, through the NF-κB signaling pathway.

Taken together, our findings partially explain the mechanism by which *H. pylori* infection influences the outcome of patients with CHD. They also suggest that the miR-25/KLF2 axis may be a potential target for *H. pylori*-associated CHD.

## Methods and Materials

### Patients and Samples

Eighty-six patients and 68 healthy subjects were enrolled in this study. All samples were collected from the 960th Hospital of Chinese PLA between June 2016 and May 2017. The protocol of the study was approved by the Ethics Review Board of the 960th Hospital of Chinese PLA, and all participants gave informed consent. The status of *H. pylori* infection among patients was evaluated using a carbon 13 urea breath test. The test was administered twice, and the presence or absence of *H. pylori* infection was confirmed in all participants within 3 months. The healthy subjects included 36 males and 22 females, with a median age of 42 years (range 28–56 years). The infected patients included 53 males and 33 females, with a median age of 40 years (23–59 years). No significant differences were noted in age or sex between the healthy subjects and patients. Cell-free plasma was isolated and stored as previously described (Li et al., 2012).

### Exosome Purification and Electron Microscopy

For isolation of exosomes from the supernatant of GES-1 cells, Dulbecco's Modified Eagle's medium (DMEM)/high glucose medium was supplemented with 10% exosome-depleted fetal bovine serum (FBS) (prepared by overnight ultracentrifugation at 100,000 × *g* at 4°C). Exosomes were isolated by ultracentrifugation at 110,000 × g for 70 min and washed with phosphate-buffered saline (PBS). The pelleted exosomes were resuspended in 100 μL PBS. For isolation of the exosomes from plasma, a Total Exosome Isolation Kit (for plasma) (ThermoFisher, Shanghai, China) was used according to the manufacturer's protocol. For examination by electron microscopy, exosomes were fixed with 2% paraformaldehyde, loaded onto 200-mesh Formvar-coated grids, after which they were contrasted and embedded, as described in a previous study (Thery et al., 2006).

### RNA Extraction and Quantitative Reverse-Transcriptase Polymerase Chain Reaction (qRT-PCR)

The TRIzol LS Reagent (Thermo Fisher Scientific, Shanghai, China) was used to extract total RNAs from 250 μL plasma according to the manufacturer's instructions. To normalize the expression of miR-25, 10 μL of 0.05 μM *Caenorhabditis elegans* miR-39 (cel-miR-39) (GenePharma, Shanghai, China) was added to the 250 μL of plasma. The TRIzol Reagent (Thermo Fisher Scientific) was used to isolate total RNAs from GES-1 cells, according to the manufacturer's protocol.

Reverse transcription of miR-25 and cel-miR-39 was performed using a TaqMan microRNA Reverse Transcription Kit (Applied Biosystems, Foster City, CA, USA), and a PrimeScript RT reagent Kit (TaKaRa, Dalian, China) was used for mRNAs, according to the manufacturer's instructions. The synthesized cDNAs were amplified in a StepOnePlus™ Real-Time PCR System using a Premix Ex Taq Kit (TaKaRa) according to the manufacturer's protocol. Parameters of the PCR reaction were as follows: 95°C for 1 min, 40 cycles at 95°C for 15 s, and 60°C for 30 s. The sequences of the primers used are presented in Table S1. These experiments were repeated three times.

### Immunoblotting

Total proteins from exosomes or cells were isolated using the RIPA Lysis and Extraction Buffer (Invitrogen) according to the manufacturer's protocol. The proteins were subjected to 10% sodium dodecyl sulfate-polyacrylamide gel electrophoresis (SDS-PAGE), after which they were transferred onto polyvinylidene difluoride (PVDF) membranes (Millipore, Massachusetts, USA), and then blocked with 5% skim milk. The PVDF membranes were incubated with VCAM-1 (Cell Signaling Technology, CST, Boston, USA, #32653); ICAM-1 (CST, #4915); CD63 (Abcam, Shanghai, China, ab134045); KLF2 (Abcam, ab203591); or GAPDH (CST, #5174) antibodies overnight at 4°C, and then incubated with secondary antibodies (goat anti-rabbit or mouse) (ZSGB-BIO, Beijing, China) for 1 h. The PVDF membranes were then exposed in a chemiluminescence instrument (Bio-Rad ChemiDoc XRS+, USA) using the SuperSignal West Dura Extended Duration Substrate Kit (Thermo, Scientific, Beijing, China).

### Cell and Bacterial Culture

The GES-1 and HEK293 cell lines were purchased from the American Type Culture Collection (ATCC). Both cell lines were cultured with DMEM/high glucose medium (HyClone, Logan, UT, USA) supplemented with 10% fetal bovine serum (Gibco, USA) at 37°C in 5% CO_2_. The *H. pylori* strain 11637 was purchased from the ATCC. Bacteria were grown on CDC blood agar plates (BD Biosciences, USA) at 37°C for 2 days in an anaerobic jar containing a gas mixture of 5% O_2_, 10% CO_2_, and 85% N_2_. *H.pylori* was added to GES-1 cells at a multiplicity of infection of 100:1.

### Vector Construction

To construct the KLF2 3′UTR reporter vectors, the DNA sequence encompassing the putative miR-25 binding sites or a scrambled control sequence was inserted into the *Spe* I/*Hind* I sites of the pMIR-REPORT luciferase vector (Promega; Madison, WI, USA). For KLF2 overexpression, the *homo sapiens* full open reading frame cDNA clone of KLF2 was inserted into a pcDNA3.1 vector (Invitrogen). The IgK-IFN-luc vector (Addgene, USA) was used to detect NF-κB activation.

### Luciferase Assay

Cells were grown up to 80% confluence in 24-well plates and transfected with the IgK-IFN-luc vector, pMIR-REPORT luciferase vector, Renilla luciferase control vector (pRL-TK, Promega), and/or miR-25 mimics using lipofectamine 2000 (Thermo Fisher Scientific). Twenty-four hours after transfection, luciferase activity was measured using the luciferase assay substrate (Promega) according to the manufacturer's protocol. Luciferase activity was normalized to pRL-TK. These experiments were repeated three times.

### Statistical Analysis

All data were presented as the mean ± standard deviation. The two–tailed Student's *t*-test and non-parametric Mann–Whitney–Wilcoxon test were used to analyze the differences between two groups. The one-way ANOVA was used to compare the differences among more than two groups, followed by the Tukey's *post hoc* test. A P <0.05 was considered statistically significant. All statistical analyses were performed using the GraphPad Prism 8.0 (GraphPad Software Inc, CA, USA) and SPSS 19.0 software (Chicago, IL, USA).

## Data Availability Statement

All datasets generated for this study are included in the manuscript/Supplementary Files.

## Ethics Statement

The studies involving human participants were reviewed and approved by the Ethics Review Board at the 960th Hospital of Chinese PLA. The patients/participants provided their written informed consent to participate in this study.

## Author Contributions

NL and TW designed this study. NL, SL, KD, and GZ performed the experiments. JH and ZW conducted the statistical analysis. NL and TW drafted the article. All authors gave final approval of the version to be submitted.

### Conflict of Interest

The authors declare that the research was conducted in the absence of any commercial or financial relationships that could be construed as a potential conflict of interest.
