# Supplementary figures and images for: Exosome-Transmitted miR-25 Induced by H. pylori Promotes Vascular Endothelial Cell Injury by Targeting KLF2
Source: Front Cell Infect Microbiol. 2019 Oct 29;9:366. doi: 10.3389/fcimb.2019.00366 (PMC6842922; doi:10.3389/fcimb.2019.00366)

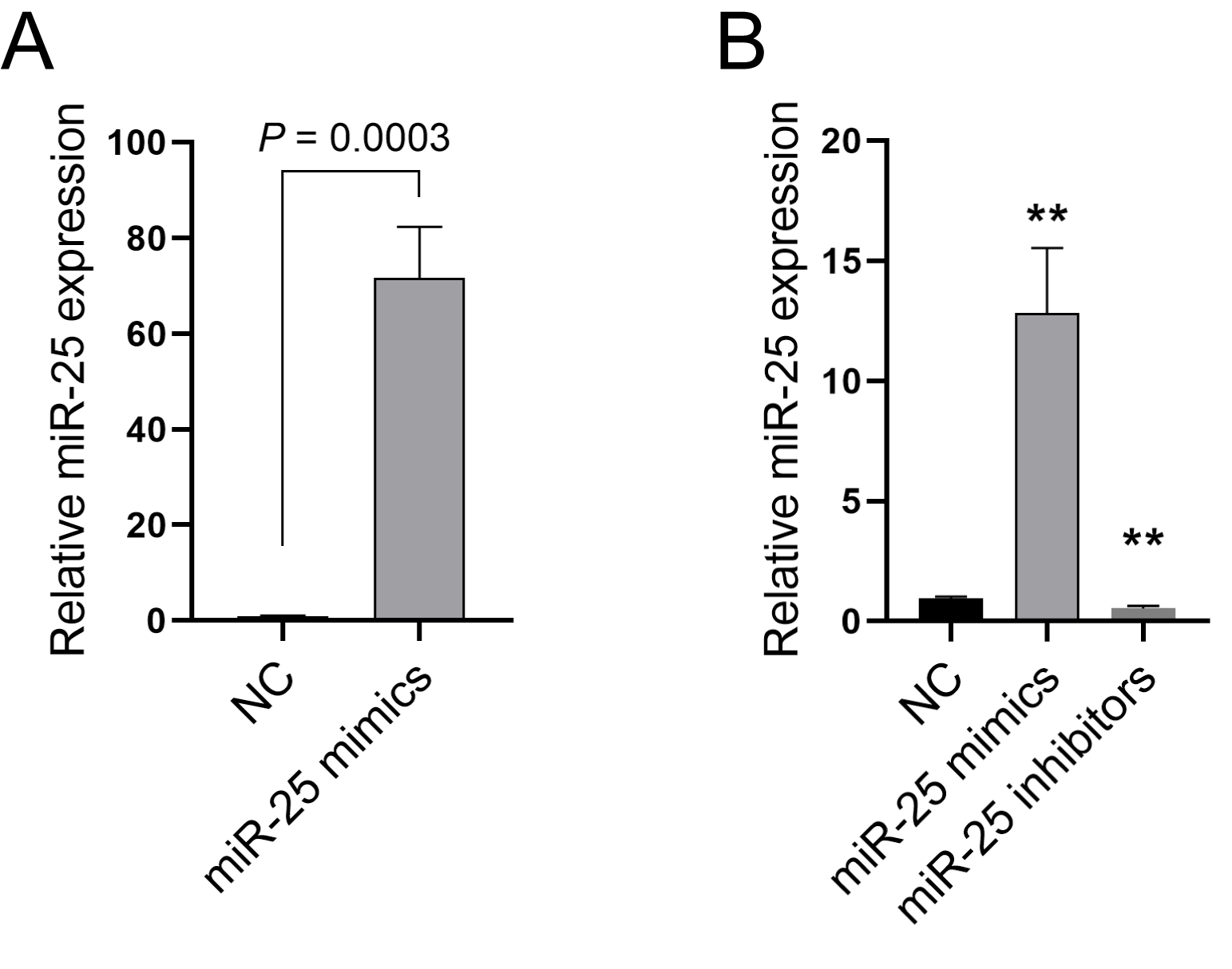

Supplement: Supplementary file 1 [file Image_1.TIF]

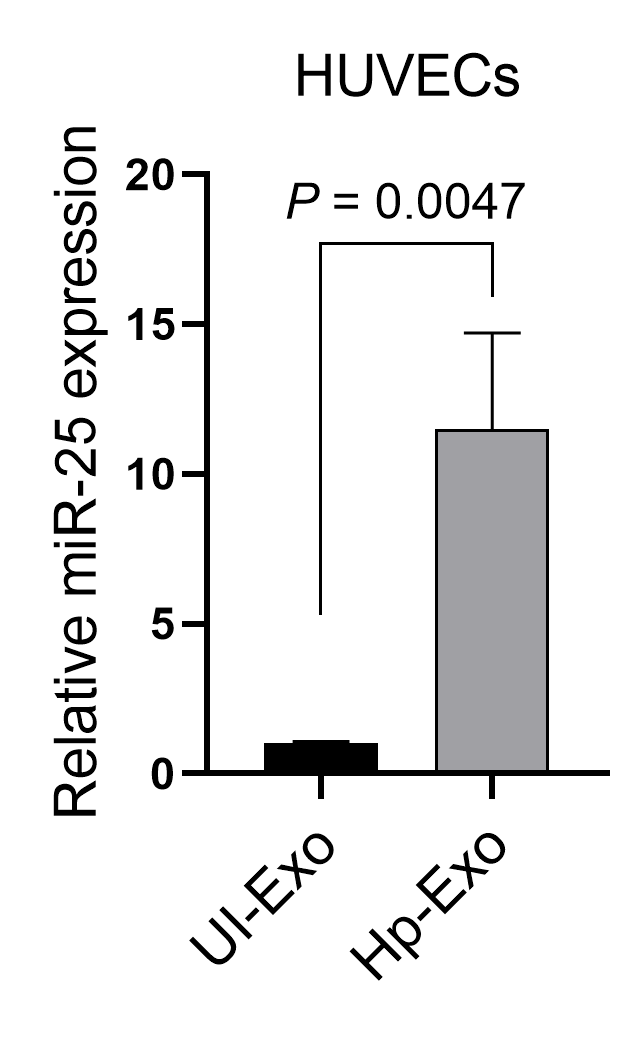

Supplement: Supplementary file 2 [file Image_2.TIF]
