# Supplementary material for: Exosome-Transmitted miR-25 Induced by H. pylori Promotes Vascular Endothelial Cell Injury by Targeting KLF2
Source: Front Cell Infect Microbiol. 2019 Oct 29;9:366. doi: 10.3389/fcimb.2019.00366 (PMC6842922; doi:10.3389/fcimb.2019.00366)
Supplement: Supplementary file 3 [file Table_1.DOCX]

**Supplementary data**

**Figure legends**

**Figure S1. Identification of effect of miR-25 mimics and inhibitors.** **(A)** Bars show the expression of miR-25 in HUVECs which were transfected with miR-25 mimics (200 nM), detected by qRT-PCR, U6 as the internal reference. (B) Bars show the expression of miR-25 in GES-1 cells which were transfected with miR-25 mimics (200 nM) or inhibitors (200 nM), detected by qRT-PCR, U6 as the internal reference.

Figure S2. The expression of miR-25 in HUVECs which incorporated the exosomes derived from GES-1 with or without *H. pylori* infection, detected by qRT-PCR, U6 as the internal refernce.

Table S1

| VCAM-1 forward: | TCTGTGACCATGACCTGTTCCA |
| --- | --- |
| VCAM-1 reverse: | TCTCCAATCTGAGCAGCAATCC |
| ICAM-1 forward: | TGCCTTGTCCTCTTGTCCTGTT |
| ICAM-1 reverse: | TGTGGTGTTGTGAGCCTATGGT |
| IL-6 forward: | CCTTCGGTCCAGTTGCCTTCT |
| IL-6 reverse: | GAGGTGAGTGGCTGTCTGTGT |
| MCP-1 forward: | CTTCTGTGCCTGCTGCTCATAG |
| MCP-1 reverse: | GACACTTGCTGCTGGTGATTCT |
| KLF2 forward: | CGGCAAGACCTACACCAAGAGT |
| KLF2 reverse: | CGCACAGATGGCACTGGAATG |
| GAPDH forward: | GGTGGTCTCCTCTGACTTCAACA |
| GAPDH reverse: | TCTCTTCCTCTTGTGCTCTTGCT |
